# Ketamine improves ovarian function and oocyte quality in CORT-induced depression mouse model by ferroptosis pathway

**DOI:** 10.1038/s41420-026-03291-9

**Published:** 2026-08-08

**Authors:** Yuqing Liu, Jiang Jiang, Yalong Wang, Zhaocheng Zeng, Liying Zhou, Zhiqin Li, Bin Deng, Jinling Wang, Hailong Wang

**Affiliations:** 1 Department of Basic Medicine, School of Medicine, Xiamen University, Xiamen, China; 2 Reproductive Medical Center, Zhanjiang Jiuhe Hospital, Zhanjiang, China; 3 Center for Reproductive Medicine, Maternity and Child Health Care Hospital in Xiangtan, Xiangtan, China; 4 Reproductive Medicine Center, Xiamen University Affiliated Chenggong Hospital, Xiamen, China; 5 Department of Anesthesiology, Xiang’an Hospital of Xiamen University, School of Medicine, Xiamen University, Xiamen, China; 6 Department of Anesthesiology, The First Affiliated Hospital of Xi’an Jiaotong University, Xi’an, China; 7 Department of Emergency and Critical Care Center, The Second Affiliated Hospital of Guangzhou University of Chinese Medicine, Zhanjiang, China

**Keywords:** Reproductive disorders, Depression

## Abstract

Depression is a common psychiatric disorder, with a higher prevalence in women than in men, and has been increasingly associated with disturbances in female reproductive health. In particular, depressive conditions may impair ovarian function and compromise oocyte quality, thereby contributing to reproductive disorders. Ketamine is a clinically effective antidepressant. However, its potential role in improving depression-induced female reproductive disorders remains unclear. In the present study, a corticosterone (CORT)-induced mouse model of depression was used to evaluate whether ketamine could ameliorate ovarian dysfunction and oocyte impairment. Ketamine treatment significantly improved depressive-like behaviors and partially reversed abnormalities in ovarian function and oocyte quality. Metabolomics and single-cell transcriptomics analyses suggested that ketamine may enhance oocyte quality in depressive mice through the regulation of ferroptosis. Our findings confirmed that ketamine ameliorated alterations in ROS, Fe^2+^, glutathione (GSH), and lipid peroxidation levels in oocytes, reduced ovarian mitochondrial abnormalities, and regulated the ferroptosis-related proteins GPX4, FTH1, and FTL in ovarian tissues. In conclusion, ketamine alleviates depression-associated ovarian dysfunction and oocyte impairment, potentially through modulation of ferroptotic signaling, offering new insights linking mental disorders to female reproductive health.

**Figure:**
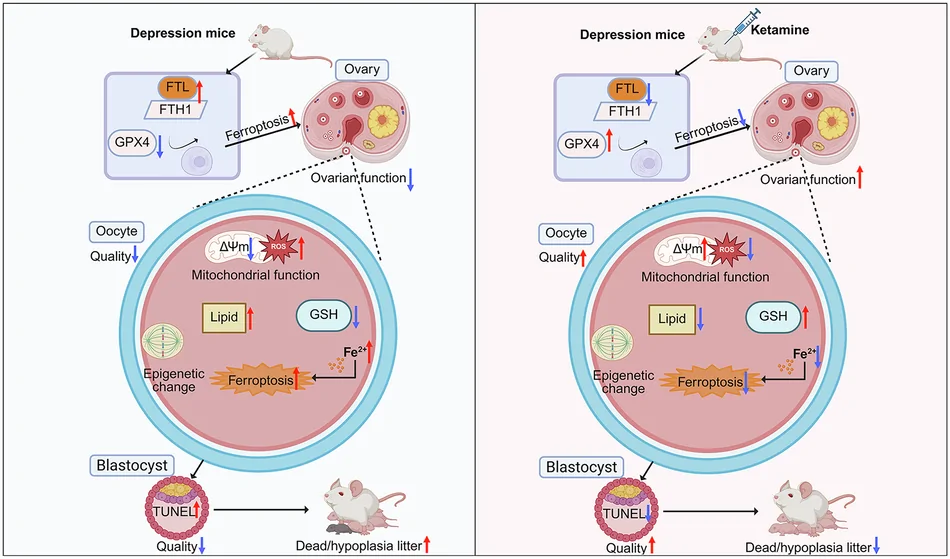

## Introduction

Depression is a mood disorder characterized by a long-lasting low mood and/or an inability to experience pleasure [1]. It represents a leading cause of global disability and lost productive years, according to the World Health Organization, which estimates that approximately 332 million individuals are affected worldwide [2]. The incidence of depressive disorders has risen significantly, with major depressive disorder impacting young populations more severely [3]. Beyond its psychological manifestations, depression disrupts multiple physiological systems, including the reproductive axis, causing female reproductive health abnormalities [4]. Women are about 1.5 times more likely to experience depression than men [2]. Depression and infertility have a bidirectional relationship [5, 6], with infertile women having a higher prevalence of depressive symptoms than women without reproductive impairment [7]. Growing evidence links ovarian disorders to the onset or exacerbation of mood disturbances, underscoring the reciprocal interaction between reproductive and mental health [8, 9].

The ovary is central to female reproductive capacity, coordinating folliculogenesis, oocyte maturation, and endocrine signaling. Oocyte quality determines fertilization efficiency, embryonic development potential, and pregnancy outcomes [10]. A long-term depressive state affects hypothalamic-pituitary-adrenal (HPA) axis function, thereby disrupting hypothalamic-pituitary-ovarian (HPO) axis activity. Dysregulated secretion of gonadotropic and stress-related hormones interferes with ovarian endocrine homeostasis, disrupts follicular development, and affects the cellular and metabolic integrity of oocytes [11]. Rising work pressure and environmental changes have contributed to a growing incidence of female reproductive disorders. Meanwhile, women with reproductive diseases face a higher risk of comorbid mental disorders [12]. These processes could impair fertilization and embryonic development, and even affect the offspring’s health.

Ketamine, a classic N-methyl-D-aspartate receptor antagonist [13], exerts antidepressant effects by modulating glutamatergic signaling, enhancing neuroplasticity, and regulating inflammatory responses [14]. Ketamine influences cellular redox and death pathways, including ferroptosis, by promoting neuroplastic adaptations, stimulating autophagic processes, and limiting ferroptotic injury within the habenular nucleus, mechanisms that may contribute to its rapid therapeutic action [15]. Ketamine alleviates ferroptosis-mediated acute lung injury and myocardial injury [16, 17]. This provides a novel perspective on its antidepressant mechanisms.

Ferroptosis involves iron-dependent lipid peroxidation, a feature that distinguishes the process and ultimately leads to programmed cell death [18, 19]. Patients with depressive disorders present ferroptosis-related pathological changes, including disturbances in iron homeostasis and increased lipid peroxidation. These observations indicate that ferroptotic signaling participates in the onset and progression of depression [20–23]. Ferroptosis is critical in the pathological processes of female reproductive disorders. Studies have confirmed that depression and chronic psychological stress are closely associated with female ovarian diseases [24, 25]. Stress and depressive states can induce ovarian iron metabolism disorder, lipid homeostasis imbalance, and oxidative damage, thereby promoting granulosa cell death, disrupting normal follicular development, causing reproductive hormone dysregulation, and ultimately resulting in both structural and functional impairment of the ovary [26–28].

Prior studies have separately linked depression with compromised oocyte quality, established the rapid antidepressant efficacy of ketamine, and implicated ferroptosis in related diseases. However, whether ketamine may improve depression-induced ovarian dysfunction and oocyte quality impairment remains unclear. This study investigates the potential protective effects of ketamine on ovarian dysfunction and impaired oocyte competence in a mouse model of CORT-induced depression and aims to determine whether these effects are mediated by the ferroptosis pathway.

## Results

### Effects of ketamine on behavioral tests in CORT-induced depression mouse model

**Fig. 1:**
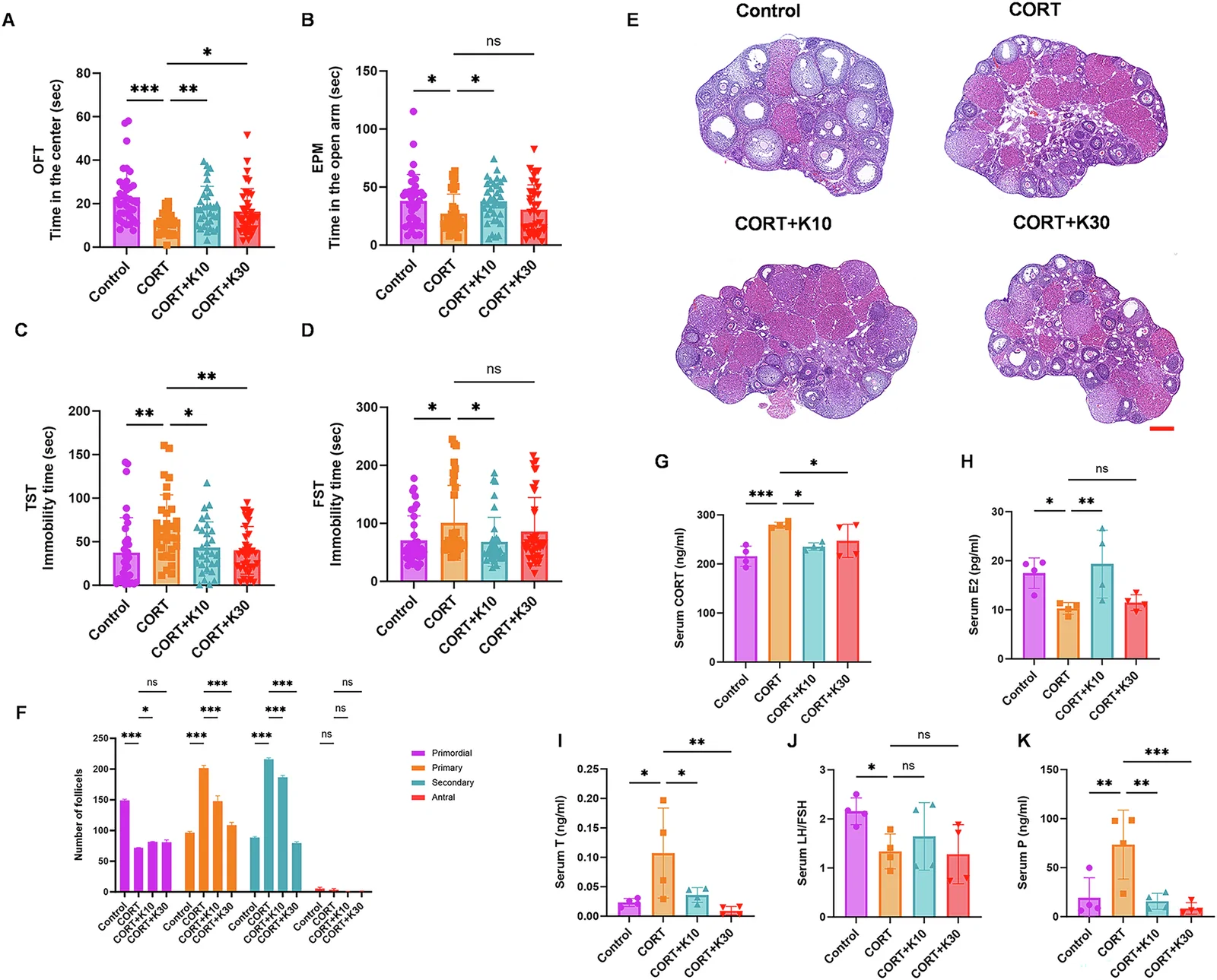
Effects of ketamine on behavioral tests and follicular development in the CORT-induced depression mouse model. **A** Statistical analysis of the time spent in the central area of mice in OFT (*n* = 37). **B** Statistical analysis of time spent in the open arms of mice in EPM (*n* = 37). **C**, **D** Statistical analysis of immobility time of mice in TST and FST (*n* = 37). **E** Representative HE-stained ovarian sections. Scale bar = 200 μm. **F** Statistical analysis of the number of follicles at different stages (*n* = 3). **G**–**K** Statistical analysis of serum levels of CORT, E2, T, LH/FSH, and P (*n* = 4). *P < 0.05, **P < 0.01, ***P < 0.001.

Behavioral tests were conducted. In the open field test (OFT) (Fig. 1A), the CORT group spent significantly less time in the central area compared with the control. Both the CORT + K10 and CORT + K30 groups spent significantly more time in the central area than the CORT group. In the elevated plus maze (EPM) (Fig. 1B), the CORT group showed a marked reduction in the duration spent in the open arms compared with the control. The CORT + K10 group significantly increased open-arm exploration compared with the CORT group, whereas the CORT + K30 group did not differ significantly from the CORT group. In both the tail suspension test (TST) and the forced swimming test (FST), immobility time was significantly longer in the CORT group compared with the control, whereas the CORT + K10 group showed a significant reduction compared with the CORT group (Fig. 1C, D).

### Effects of ketamine on follicular development in CORT-induced depression mouse model

Body weight was monitored every 3 days (Fig. S1B). Over the 21-day experimental period, the CORT group showed significantly reduced body weight gain compared with the control group. Both CORT + K10 and CORT + K30 groups showed a declining trend in body weight gain. Analysis of ovarian coefficients revealed no significant differences (Fig. S1C). Statistical comparisons did not reveal significant differences in the examination of estrous cycle stages (Fig. S1D, E).

After HE staining, follicles at various stages were counted (Fig. 1E, F). The CORT group exhibited fewer primordial follicles than the control. The number of primordial follicles in the CORT + K10 group was higher compared with the CORT group, whereas the CORT + K30 group did not differ significantly from the CORT group. The CORT group had more primary and secondary follicles than the control. Both ketamine-treated groups had fewer primary and secondary follicles compared with the CORT group. No significant differences in antral follicle numbers were observed.

Hormonal analysis revealed notable endocrine alteration (Fig. 1G–K). Compared with the control group, mice in the CORT group showed elevated serum corticosterone, testosterone (T), and progesterone (P) levels, while the ratio of luteinizing hormone (LH)/follicle-stimulating hormone (FSH) and estradiol (E2) levels decreased. In the CORT + K10 group, compared with the CORT group, serum concentrations of corticosterone, T, and P were decreased, whereas E2 levels were increased. Nevertheless, the LH/FSH ratio remained dysregulated. In the CORT + K30 group, serum concentrations of T and P were lower than those in the CORT group, while the other hormones remained dysregulated.

### Effects of ketamine on in vitro and in vivo oocyte maturation in the CORT-induced depression mouse model

**Fig. 2:**
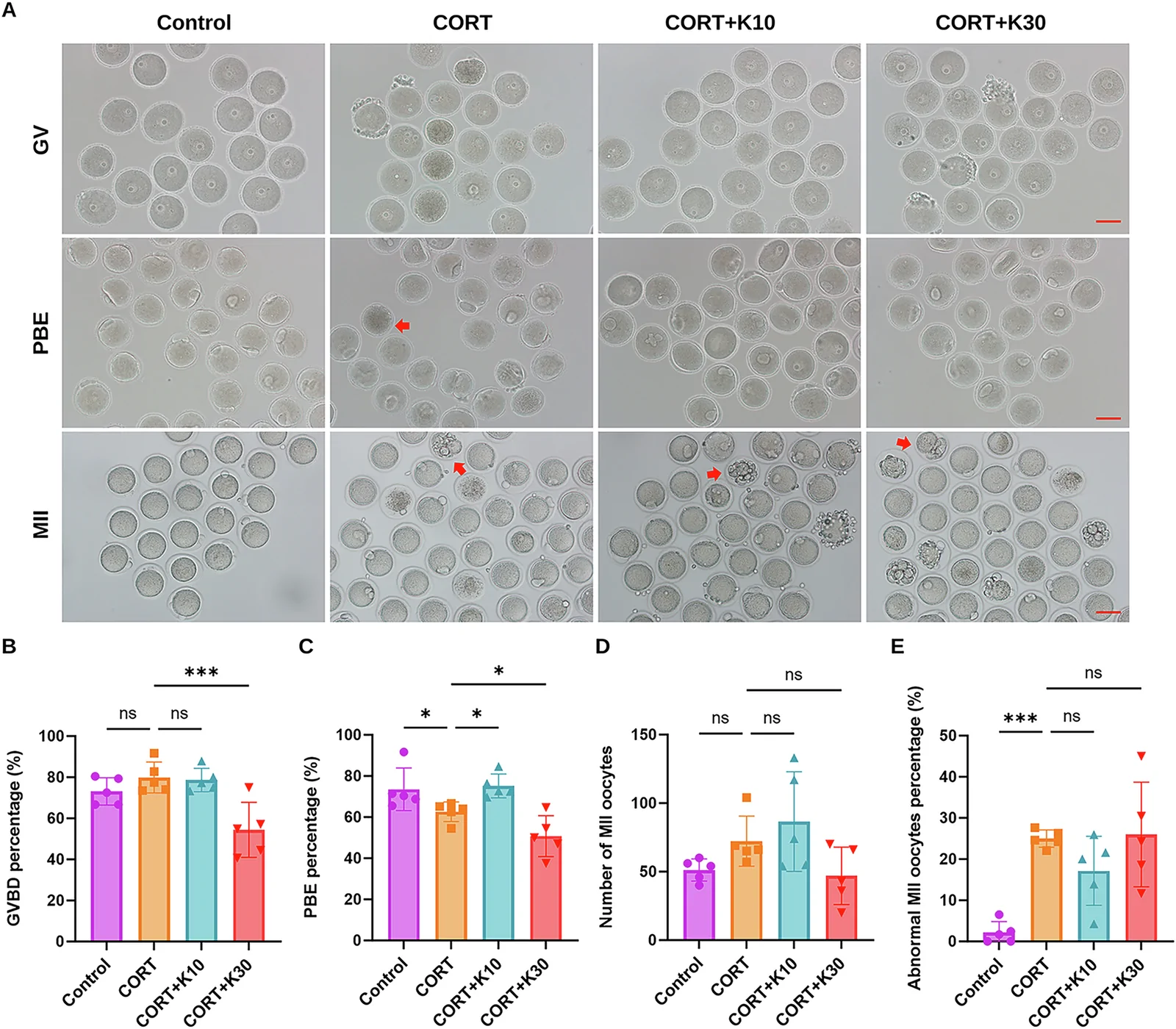
Effects of ketamine on in vitro and in vivo oocyte maturation in the CORT-induced depression mouse model. **A** GV-stage oocytes from the ovaries of each group. GV-stage oocytes cultured for 12 h until polar body extrusion. MII-stage oocytes from the oviducts of each group. Scale bar = 100 µm. Red arrow: abnormal oocytes. **B** Statistical analysis of the GVBD rate (*n* = 5). **C** Statistical analysis of the polar body extrusion rate (*n* = 5). **D** Statistical analysis of the number of MII oocytes (*n* = 5). **E** Statistical analysis of the abnormal MII oocyte rate (*n* = 5, each replicate contained oocytes from two mice). Scale bar = 100 µm. *P < 0.05, **P < 0.01, ***P < 0.001.

Following superovulation, germinal vesicle (GV) stage oocytes were collected and cultured in vitro. After 2 h of culture, germinal vesicle breakdown (GVBD) rates were recorded (Fig. 2A, B). No statistically significant differences in GVBD rates were detected between the CORT and control groups or between the CORT and CORT + K10 groups. After 12 h of culture, the results showed that the CORT group had a significantly lower first polar body extrusion (PBE) rate than the control group (Fig. 2A, C). This reduction was significantly alleviated in the CORT + K10 group. The PBE rate in the CORT + K30 group significantly decreased.

In vivo mature metaphase II (MII) oocytes were also collected. The number of MII oocytes retrieved from the CORT group did not differ significantly from that of the control group (Fig. 2D). However, the proportion of abnormal MII oocytes was significantly higher in the CORT group than in the control group (Fig. 2E). The number of MII oocytes retrieved in the CORT + K10 group did not differ significantly from that of the CORT group, while its proportion of abnormal MII oocytes showed a decreasing trend.

### Effect of ketamine on oocyte oxidative damage and apoptosis in CORT-induced depression mouse model

**Fig. 3:**
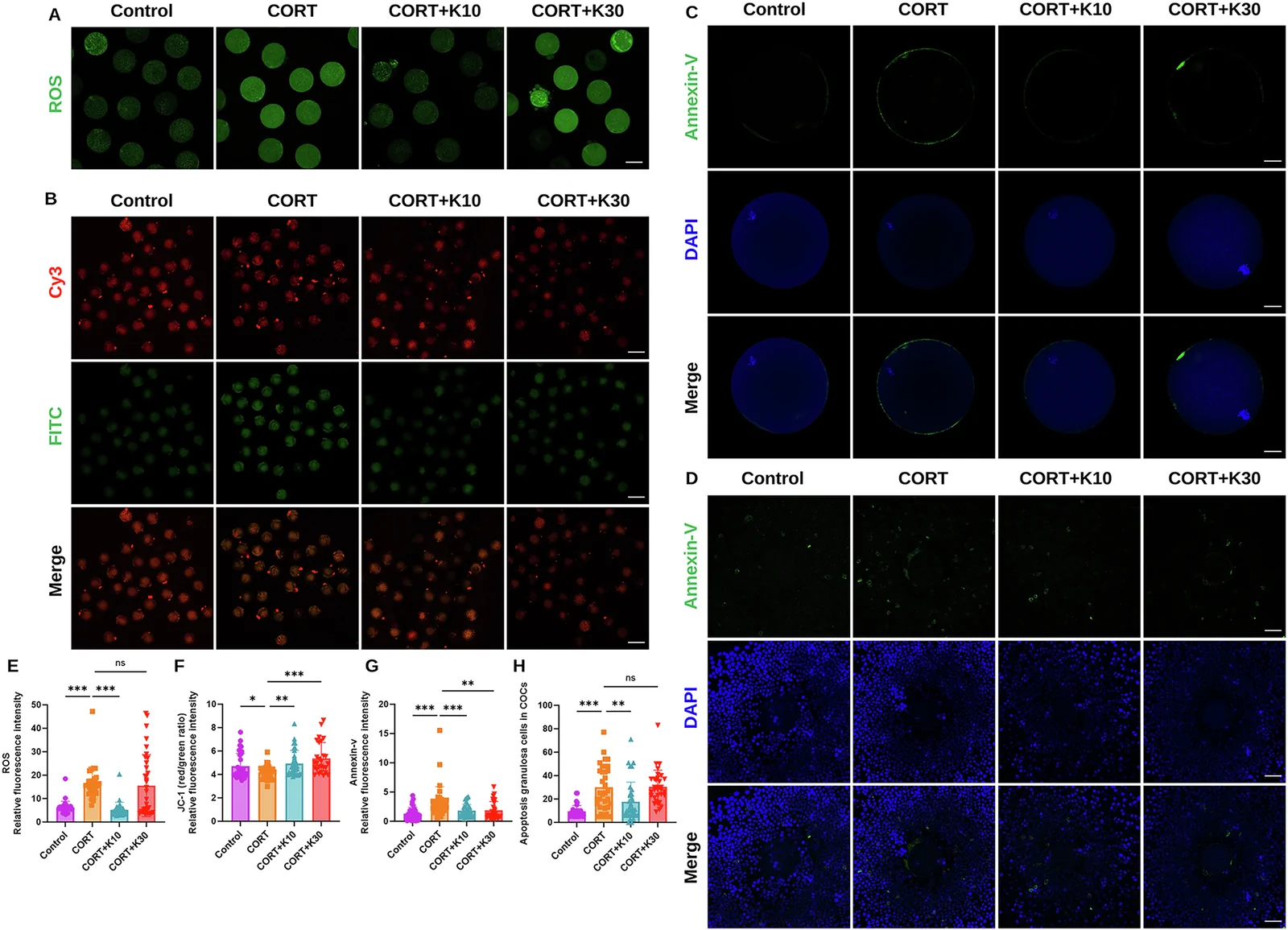
Effect of ketamine on oocyte oxidative damage and apoptosis in the CORT-induced depression mouse model. **A** Representative images of ROS signals in MII oocytes. ROS, green. Scale bar = 50 μm. **B** Representative images of JC-1 staining of MII oocytes. High ΔΨm, red; Low ΔΨm, green. Scale bar = 100 μm. **C** Representative images of apoptosis (Annexin-V) signals in MII oocytes. Annexin-V, green; DAPI, blue. Scale bar = 20 μm. **D** Representative images of Annexin-V signal in cumulus granulosa cells. Annexin-V, green; DAPI, blue. Scale bar = 50 μm. **E** Statistical analysis of ROS fluorescence intensity in oocytes from each group (*n* = 32/31/31/35). **F** Statistical analysis of JC-1 fluorescence intensity ratio (red/green) in oocytes (*n* = 33/34/34/27). **G** Statistical analysis of Annexin-V fluorescence intensity in the oocytes (*n* = 35/36/36/30). **H** Statistical analysis of the number of apoptotic granulosa cells in COCs (*n* = 34/33/37/33). *P < 0.05, **P < 0.01, ***P < 0.001.

ROS levels were quantified in MII oocytes (Fig. 3A, E). The results showed that ROS levels in the CORT group were significantly higher than those in the control group. Compared with the CORT group, the CORT + K10 group exhibited a clear reduction in ROS accumulation, whereas the CORT + K30 group did not show a statistically significant. To further assess mitochondrial function, JC-1 staining was performed to detect mitochondrial membrane potential in MII oocytes (Fig. 3B, F). The mitochondrial membrane potential in the CORT group was lower than in the control group. Both the CORT + K10 and CORT + K30 groups demonstrated increased mitochondrial membrane potential relative to the CORT group.

Apoptotic signaling in MII oocytes was evaluated by measuring the relative fluorescence intensity of Annexin-V (Fig. 3C, G). The apoptotic signal in the CORT group was significantly higher than that of the control, and was significantly reduced in the CORT + K10 group than in the CORT group. Cumulus granulosa cells within COCs that exhibited a clear apoptotic signal were also quantified (Fig. 3D, H). The CORT group showed a marked presence of apoptotic cumulus granulosa cells compared with the control group, and 10 mg/kg ketamine significantly reduced the number of apoptotic cells induced by CORT exposure.

### Effects of ketamine on oocyte cytoskeleton in CORT-induced depression mouse model

**Fig. 4:**
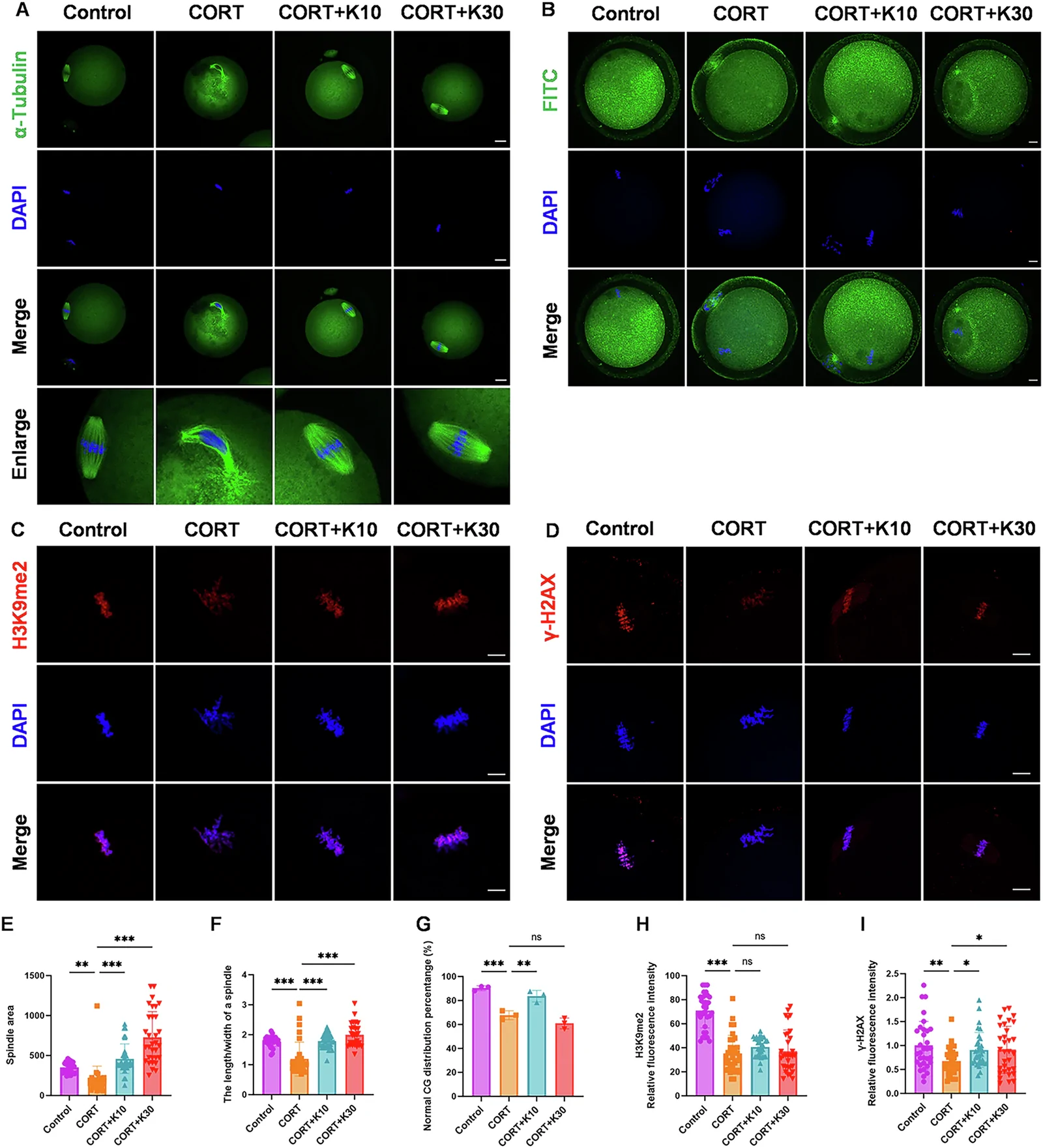
Effects of ketamine on oocyte cytoskeleton, DNA damage, and epigenetic changes in the CORT-induced depression mouse model. **A** Representative images of spindle morphology and chromosomes alignment of MII oocytes. α-tubulin, green; DAPI, blue. Scale bar = 20 μm. **B** Representative immunofluorescent images of cortical granules (CGs) of MII oocytes, scale bar = 20 µm. A normal MII oocyte develops a cortical region near the spindle, and its cells exhibit polarity. **C** Representative images of H3K9me2 staining of MII oocytes. H3K9me2, red; DAPI, blue. Scale bar = 10 μm. **D** Representative images of γ-H2AX staining of MII oocytes. γ-H2AX, red; DAPI, blue. Scale bar = 10 μm. **E** Statistical analysis of spindle area (*n* = 33/32/33/32). **F** Statistical analysis of the spindle length/width (*n* = 30/32/32/30). Spindle length and width were measured using ZEN software. **G** Statistical analysis of the percentage of normal CG distribution (*n* = 3). **H** Statistical analysis of H3K9me2 fluorescence intensity (*n* = 33/34/33/32). **I** Statistical analysis of the γ-H2AX fluorescence intensity (*n* = 33/34/33/32). *P < 0.05, **P < 0.01, ***P < 0.001.

We performed α-Tubulin staining on MII oocytes obtained in vivo to observe spindle morphology (Fig. 4A, E, F). Statistical analysis showed that the spindle area was smaller in the CORT group than in the control, while both the CORT + K10 and CORT + K30 groups exhibited significantly increased spindle areas compared with the CORT group. Additionally, the spindle length-to-width ratio was also calculated. The distance between the two spindle poles in the CORT group was significantly reduced relative to the control group. Both ketamine-treated groups exhibited significant improvement in this parameter compared with the CORT group.

Cortical granules (CGs) in MII oocytes were also examined (Fig. 4B, G). Normally, most cortical granules in MII oocytes are distributed polarly, forming a cortical granule-free domain around the spindle. The abnormal distribution of cortical granules was higher in the CORT group than in the control group. The abnormal proportions in the CORT + K10 group were lower than those in the CORT group.

### Effects of ketamine on DNA damage and epigenetic changes in CORT-induced depression mouse model

Immunofluorescence analysis was performed to compare the expression of histone methylation marker H3K9me2 and the DNA damage marker γ-H2AX in MII oocytes. The expression level of H3K9me2 in the CORT group was lower than that of the control. No significant differences in H3K9me2 expression were observed among the CORT, CORT + K10, and CORT + K30 groups (Fig. 4C, H).

Compared with the control group, the γ-H2AX fluorescence intensity in the CORT group was lower. Both the CORT + K10 and CORT + K30 groups showed higher γ-H2AX fluorescence intensity than in the CORT group (Fig. 4D, I). Ketamine maintained γ-H2AX expression levels in oocytes of the CORT-induced depression mouse model but failed to restore epigenetic modifications represented by H3K9me2.

### Effects of ketamine on embryonic and offspring development in the CORT-induced depression mouse model

**Fig. 5:**
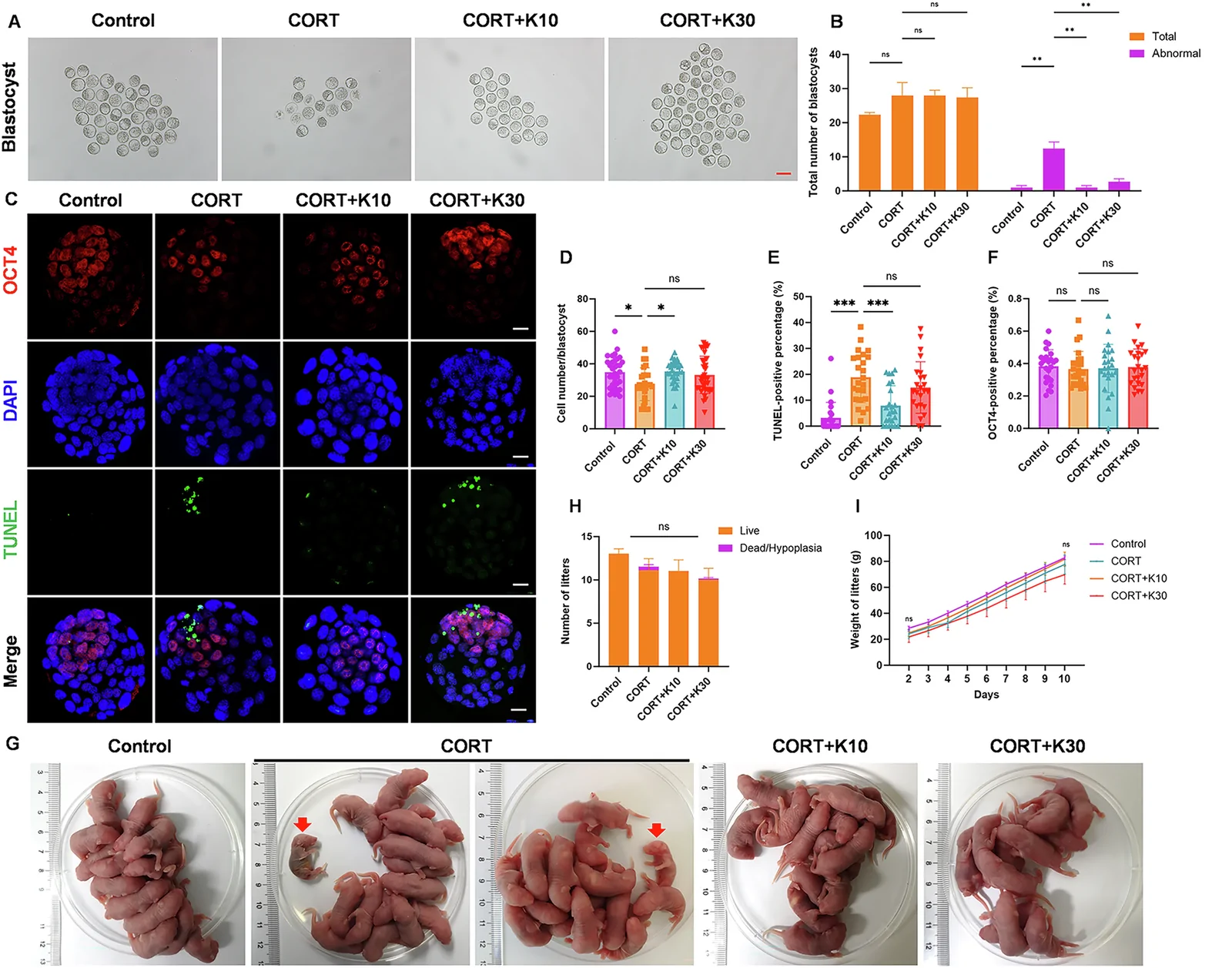
Effects of ketamine on embryonic and offspring development in the CORT-induced depression mouse model. **A** Representative images of blastocysts (3.5 dpc). Scale bar = 100 μm. **B** Statistical analysis of the total number of blastocysts and abnormal blastocysts (*n* = 3). **C** Representative images of TUNEL-positive and OCT4-positive staining. TUNEL-positive cells, green; OCT4-positive cells, red; DAPI, blue. Scale bar = 20 μm. **D** Statistical analysis of the number of cells per blastocyst (*n* = 33/25/30/35). **E** Statistical analysis of TUNEL-positive cells in blastocysts (*n* = 25). **F** Statistical analysis of OCT4-positive cells in blastocysts (*n* = 25). **G** Representative images of offspring. Red arrow: dead/hypoplastic pups. **H** Statistical analysis of litter size (*n* = 8). **I** Changes in litter weight within 10 days after birth (*n* = 5). *P < 0.05, **P < 0.01, ***P < 0.001.

Blastocysts were collected at 3.5 days post coitum (dpc) for morphological observation, and no significant difference in the total number of blastocysts obtained per mouse was observed. The CORT group had a higher number of abnormal blastocysts than the control, while both the CORT + K10 and the CORT + K30 groups had lower numbers than the CORT group (Fig. 5A, B).

To further assess the quality of mouse blastocysts, OCT4 and TUNEL staining were performed (Fig. 5C). Quantification of total cell numbers per blastocyst revealed a lower count in the CORT group than the control (Fig. 5D). The CORT + K10 group exhibited a higher cell count than the CORT group. Results showed that the CORT group had a higher proportion of TUNEL-positive cells than the control (Fig. 5E). Relative to the CORT group, the TUNEL-positive rate decreased in the CORT + K10 group, whereas no significant difference was observed in the CORT + K30 group. There was no significant difference in the OCT4-positive cell rate (Fig. 5F).

Fertility assessment showed no significant difference in litter size (Fig. 5G, H). Nevertheless, dead or hypoplastic pups were observed in the CORT and CORT + K30 groups. The offspring in the CORT + K30 group showed a trend of retarded weight gain without a significant statistical difference (Fig. 5I).

### Effects of ketamine on metabolomics in CORT-induced depression mouse model

**Fig. 6:**
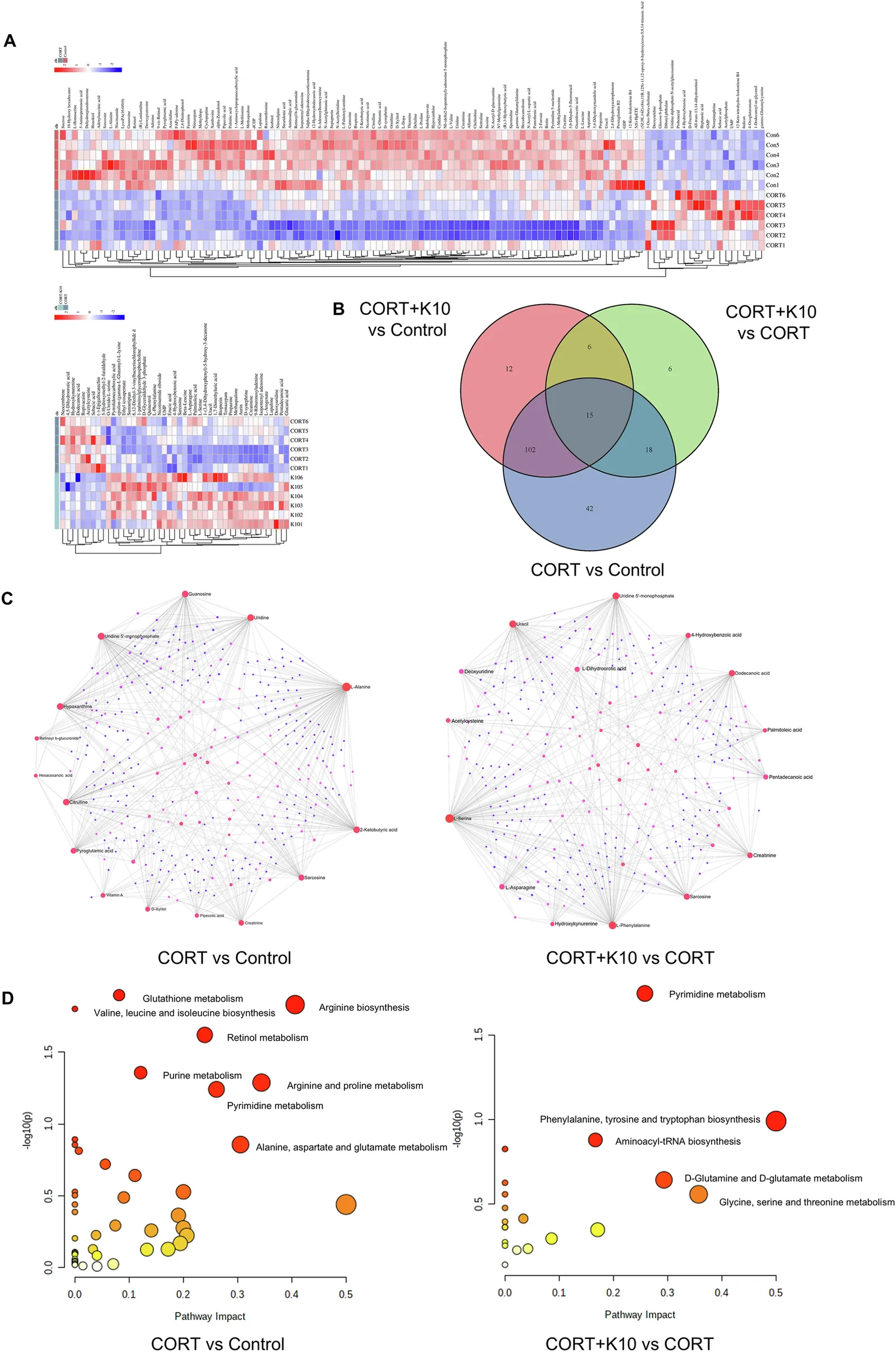
Effects of ketamine on metabolomics in the CORT-induced depression mouse model. **A** Cluster analysis heatmap of differential metabolites between the CORT and the control groups, and between the CORT + K10 and the CORT groups. **B** Venn diagram of differential metabolites among the experimental groups. **C** Central metabolic network diagram of differential metabolites between the CORT and control groups, and between the CORT + K10 and CORT groups. **D** Metabolic pathway map of enriched differential metabolites between the CORT and control groups, and between the CORT + K10 and CORT groups.

Metabolomic analysis was performed on mouse ovarian tissues. There were 177 differences in metabolites between the CORT group and the control group, with the contents of substances such as uridine, guanosine, L-alanine, creatine, creatine kinase, and cinchonine acid significantly decreased. While the differences between the CORT + K10 group and the CORT group were reflected in 45 metabolites, such as L-serine, dodecanoic acid, L-asparagine, L-phenylalanine, and biopterin, which significantly increased (Fig. 6A, B). MetaboAnalyst 5.0 analysis revealed the central network diagram of the differential metabolites between the CORT group and the control group, as well as between the CORT + K10 group and the CORT group (Fig. 6C).

KEGG pathways enrichment analysis of the differential metabolites is shown in Fig. 6D. In the comparison between the CORT and control groups, enriched metabolic pathways included arginine biosynthesis, glutathione metabolism, retinol metabolism, arginine and proline metabolism, pyrimidine metabolism, alanine, aspartate, and glutamate metabolism, among others. The differential metabolites between the CORT + K10 and the CORT groups were mainly enriched in pyrimidine metabolism, aminoacyl-tRNA biosynthesis, D-glutamine and D-glutamate metabolism, and glycine, serine, and threonine metabolism, among others.

### Effects of ketamine on transcriptomics in CORT-induced depression mouse model

**Fig. 7:**
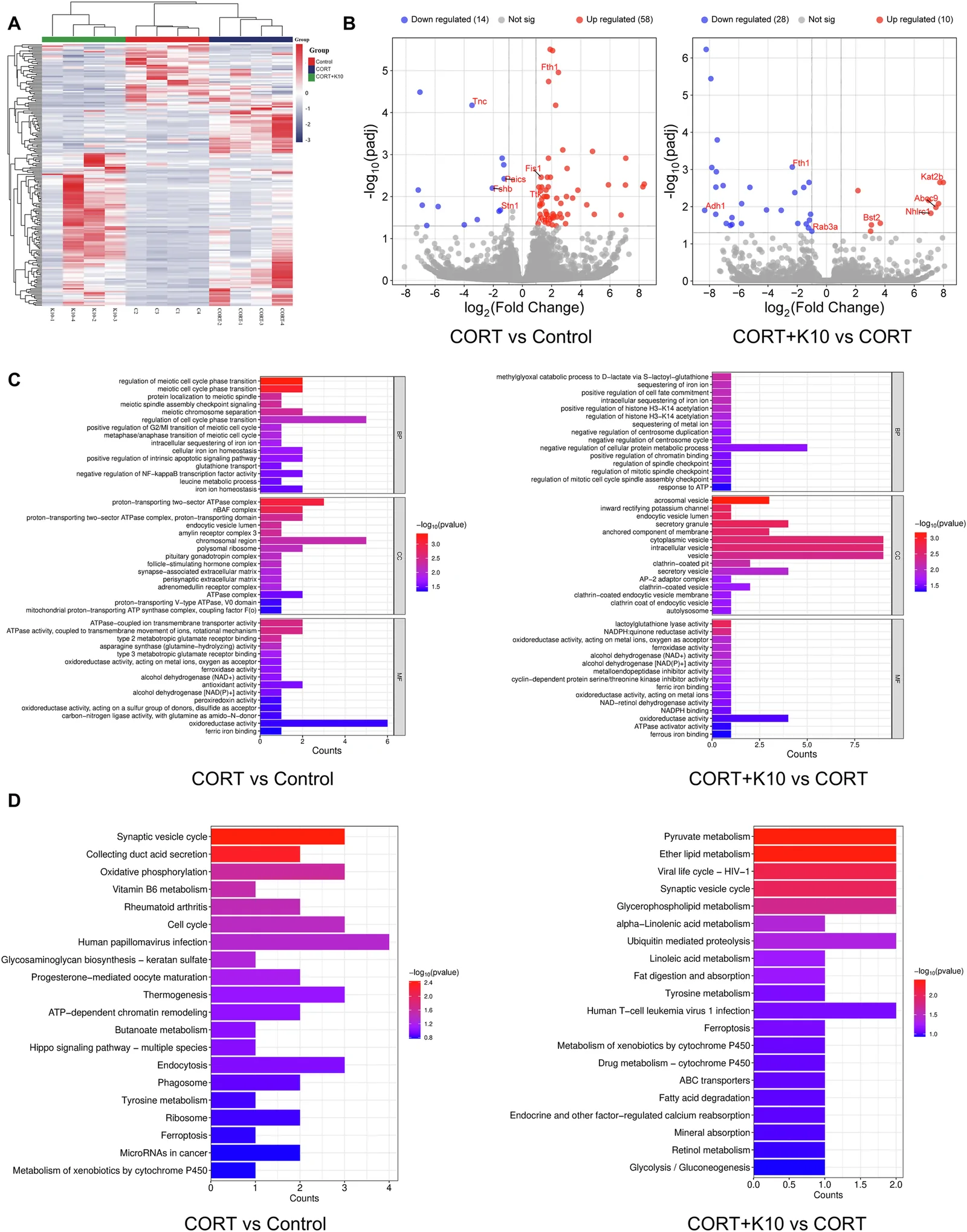
Effects of ketamine on transcriptomics in the CORT-induced depression mouse model. **A** Clustering analysis of DEGs using a heatmap. **B** Number of DEGs between the CORT and control groups, and between the CORT + K10 and CORT groups, shown in the volcano plot. **C** GO enrichment analysis of DEGs between the CORT and control groups, and between the CORT + K10 and CORT groups. **D** KEGG enrichment analysis of DEGs between the CORT and control groups, and between the CORT + K10 and CORT groups.

Single-cell transcriptomic analysis was conducted in MII oocytes. Hierarchical clustering heatmaps revealed clear differences in transcriptional profiles between the CORT and control groups, as well as between the CORT + K10 and CORT groups (Fig. 7A). A total of 72 differentially expressed genes (DEGs) were identified between the CORT and the control groups, including 58 upregulated and 14 downregulated genes. A total of 38 DEGs were identified between the CORT + K10 and the CORT groups, among which 10 were upregulated genes and 28 were downregulated genes (Fig. 7B). Gene Ontology (GO) enrichment analysis demonstrated that the DEGs between the CORT and control groups were associated with regulation of meiotic cell cycle phase transition and cellular iron homeostasis, among other biological processes (Fig. 7C). The DEGs identified between the CORT + K10 group and the CORT group were enriched in intracellular sequestering of iron and response to ATP, among others.

KEGG pathway enrichment analysis demonstrated that the DEGs between the CORT and control groups were mainly enriched in oxidative phosphorylation, cell cycle regulation, endocytosis, and ferroptosis (Fig. 7D). For the comparison between the CORT + K10 and the CORT groups, ferroptosis was also identified. The enrichment of the ferroptosis pathway, combined with iron regulation and redox reactions identified in GO analysis, suggests that ketamine may alleviate CORT-induced depression and the associated impairment of oocyte quality by regulating iron metabolism and oxidative stress during ferroptosis.

### Effects of ketamine on ferroptosis-related indicators and gene expression in the CORT-induced depression mouse model

**Fig. 8:**
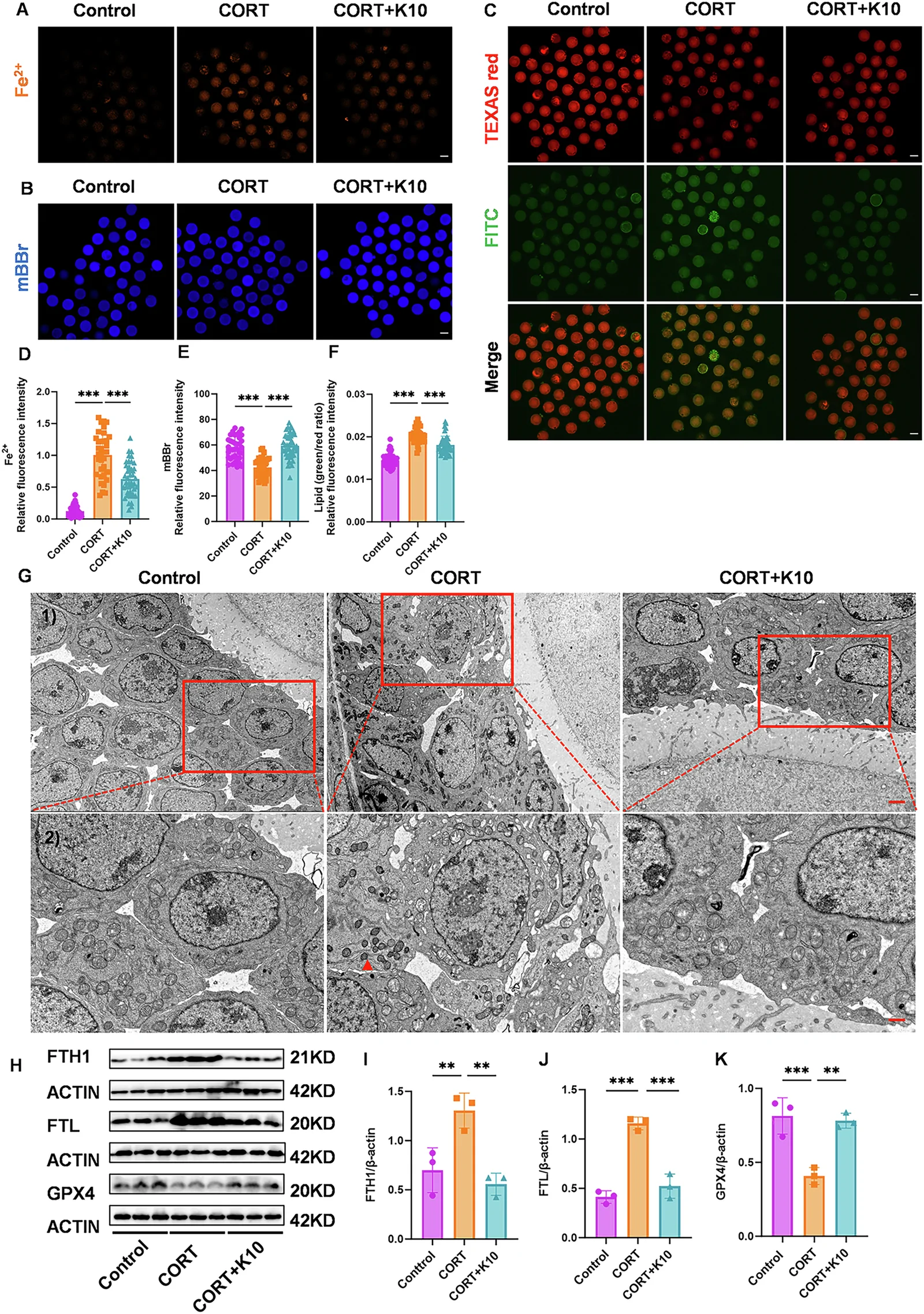
Effects of ketamine on ferroptosis-related indicators and gene expression in the CORT-induced depression mouse model. **A** Representative images of intracellular Fe^2+^ levels detected by FerroOrange staining in oocytes. Scale bar = 20 μm. **B** Representative images of GSH levels detected by mBBr staining. Scale bar = 20 μm. **C** Representative images of lipid peroxidation levels detected by C11 BODIPY 581/591 staining in oocytes (Texas Red and FITC channels). Scale bar = 20 µm. **D** Statistical analysis of fluorescence intensity of intracellular Fe^2+^ signals (*n* = 43/36/43). **E** Statistical analysis of fluorescence intensity of GSH signals (*n* = 37/42/45). **F** Statistical analysis of the ratio of green/red fluorescence intensity in each group representing the degree of lipid oxidation (*n* = 50/32/41). **G** Typical ultrastructural changes in the ovaries of each group observed with transmission electron microscopy: (1) Ultrastructure of follicles. Scale bar = 3 μm. (2) Ultrastructure of granulosa cells. Scale bar = 1 μm. Red arrows: abnormal mitochondria. **H** Western blot analysis of the changes in FTH1, FTL, and GPX4 protein expression. **I**–**K** Statistical analysis of the gray value of the western blot results (*n* = 3). *P < 0.05, **P < 0.01, ***P < 0.001.

To evaluate ferroptosis-related alterations, intracellular Fe^2+^ levels were measured in MII oocytes using FerroOrange staining (Fig. 8A, D). The Fe^2+^ level was higher in the CORT group than in the control, and significantly lower in the CORT + K10 group than in the CORT group. GSH levels were assessed using mBBr staining. GSH levels were markedly reduced in the CORT group compared with the control, whereas significantly increased in the CORT + K10 group relative to the CORT group (Fig. 8B, E). Dysregulated iron homeostasis can promote excessive ROS generation and lipid peroxidation. Compared with the control group, both ROS (Fig. 3A, E) and lipid peroxidation levels (Fig. 8C, F) were substantially elevated in the CORT group. In contrast, they were lower in the CORT + K10 group than in the CORT group.

Transmission electron microscopy (TEM) revealed notable mitochondrial structural alterations in ovaries from the CORT group, including increased mitochondrial membrane density and mitochondrial shrinkage. Some vacuolated mitochondria were present in the CORT + K10 group (Fig. 8G). Ovarian FTH1 and FTL protein levels were increased in the CORT group compared with the control, but were reduced in the CORT + K10 group relative to the CORT group (Fig. 8H–J). Ovarian GPX4 protein level was reduced in the CORT group compared with the control, and increased in the CORT + K10 group compared with the CORT group (Fig. 8H, K).

## Discussion

Depression is a prevalent psychiatric disorder that has been associated with disturbances in female reproductive health. Ketamine is a promising clinical antidepressant [29, 30]. However, whether ketamine can alleviate ovarian dysfunction and oocyte quality deterioration secondary to depressive states has not been fully clarified. Our research indicates that ketamine may partially correct abnormalities in ovarian function and oocyte competence in a CORT-induced mouse model of depression, and that this protective effect may involve modulation of the ferroptosis pathway.

Ketamine (10 mg/kg) administration improved CORT-induced depressive-like behavior, consistent with previous studies [31, 32]. Mice in the CORT group showed follicular development abnormalities and marked hormonal dysregulation, supporting the disruption of the HPO axis under depressive conditions. Chronic stress increases serum CORT levels while reducing LH, E2, and FSH levels, indicating that HPA axis abnormalities inhibit the normal function of the HPO axis [33]. Clinical and experimental evidence has associated depression with ovarian disorders, emphasizing the possibility that psychiatric conditions can directly influence reproductive physiology [9, 34, 35]. In the present study, treatment with 10 mg/kg ketamine ameliorated the abnormal follicle development observed in CORT-treated mice and partially restored serum CORT, E2, T, and P levels. Improvement of depressive-like behavior, therefore, appeared to coincide with recovery of ovarian function and normalization of endocrine activity in female mice. However, the limited efficacy observed in the 30 mg/kg ketamine group suggests a dose-dependent effect.

Improvement in depressive-like behaviors has previously been associated with enhanced oocyte quality [11, 36, 37]. The administration of ketamine (10 mg/kg) partially improved the abnormalities of oocytes. Oocyte oxidative damage and apoptosis were reduced, spindle morphology and cortical granule polarity were restored, and DNA repair capacity (as indicated by increased γ-H2AX expression) was maintained. The reduction in γ-H2AX expression in the CORT group indicates impaired DNA repair capacity, which is consistent with the reduced γ-H2AX expression in oocytes of aged mice [38]. These observations suggest that ketamine can improve CORT-induced genomic instability in oocytes. The decreased H3K9me2 levels in the CORT group indicated epigenetic modification, which aligns with previous findings that chronic stress reduced H3K9 methylation in the hippocampus of rats [39]. However, ketamine treatment did not restore H3K9me2 levels, indicating its inability to repair epigenetic disorders, while implying potential reproductive risks. Oocyte quality plays a central role in embryo development and pregnancy success, and previous studies have demonstrated that psychological stress can reduce the number of blastocysts produced and compromise their quality [40–42]. Consistent with these reports, the present study showed that treatment with 10 mg/kg ketamine improved blastocyst quality and offspring developmental outcomes.

Metabolomics profiling revealed substantial metabolic disturbances in the ovaries of the CORT-treated mice. Specifically, levels of uridine, guanosine, and alanine were significantly reduced, consistent with blood metabolites of depressed patients and other psychiatric disorder studies [43–46]. In aged mice, uridine levels decline, which in turn targets PCBP1 to trigger ferroptosis and ultimately oocyte functional deterioration [47]. The differential metabolite pathways between the CORT and control groups were associated with glutathione metabolism, purine/pyrimidine metabolism, and alanine, aspartate, and glutamate metabolism. These pathways are linked to the development of depressive disorders [48–51] and also play essential roles in ovarian follicular development and oocyte maturation [52, 53]. Among these pathways, disruption of glutathione metabolism is particularly noteworthy because it serves as a central regulatory mechanism in ferroptosis [54]. The observed metabolic alterations, therefore, provide a potential molecular connection between depressive stress and ovarian dysfunction. 10 mg/kg ketamine partially reversed CORT-induced metabolic abnormalities in mice. In particular, ovarian levels of L-serine, an amino acid involved in glutathione synthesis, were significantly increased following ketamine treatment. Levels of dodecanoic acid, a precursor that can contribute to lipid peroxidation, and hydroxykynurenine, a neurotoxic metabolite associated with the tryptophan–kynurenine pathway [55], were reduced. Biopterin is implicated in the anti-ferroptotic defense system [56]. Ketamine administration significantly rescued the CORT-induced decline of biopterin levels in the ovaries. These findings suggest that ketamine may inhibit ferroptosis by enhancing antioxidant capacity and reducing the supply of lipid peroxidation substrates [57]. Further pathway analysis comparing the CORT and CORT + K10 groups showed that the principal metabolic differences were concentrated in pyrimidine metabolism, aminoacyl-tRNA biosynthesis, and D-glutamine and D-glutamate metabolism. Previous studies have reported that ketamine pretreatment can prevent stress-induced depressive-like behavior by regulating purine and pyrimidine metabolism in the prefrontal cortex, hippocampus, and plasma of mice. Major depressive disorder is closely related to glutamate-glutamine metabolism [58–60].

The transcriptomic findings provided additional evidence for the involvement of the ferroptosis pathway in ketamine’s protective effects. DEGs between the CORT and the control groups were significantly enriched in pathways such as oxidative phosphorylation and ferroptosis, suggesting that CORT-induced depressive-like states may impair oocyte function by disrupting the balance between energy metabolism and ferroptosis. Following treatment with 10 mg/kg ketamine, enrichment analysis of DEGs revealed significant associations with functional categories including intracellular iron regulation, oxidoreductase activity, and the ferroptosis pathway. These observations indicate that ketamine may contribute to the maintenance of iron homeostasis and redox equilibrium through transcriptional regulation, which aligns with the central biological features of ferroptosis that involve disturbances in iron metabolism and lipid peroxidation.

Ferroptosis is associated with excessive iron accumulation and lipid peroxidation. Morphologically, it is characterized by pronounced mitochondrial shrinkage, condensed membrane density, and reduction or loss of mitochondrial cristae [61]. Ovarian mitochondrial ultrastructural changes in the CORT group reflected these characteristics. In the CORT + K10 group, slight mitochondrial vacuolation occurred, rather than the typical mitochondrial shrinkage. Ferroptosis is also characterized by elevated free iron and accumulation of intracellular ROS and lipid peroxides. Iron accumulation, a key hallmark of ferroptosis, promotes lipid peroxidation through Fenton-like reactions, while intracellular GSH depletion impairs lipid peroxide metabolism, further facilitating ferroptosis [18]. Ketamine inhibited the accumulation of Fe^2+^, ROS, and lipid peroxides, and restored GSH content in oocytes of depressive mice. This implies that ketamine’s functional effects may occur through its intervention in the ferroptosis pathway.

Dysfunction of GPX4 leads to lipid peroxidation, as it employs glutathione to convert toxic lipid peroxides into non-toxic lipid alcohols, preventing iron-dependent cell death [62]. The majority of intracellular iron is sequestered in ferritin, a protein complex composed of ferritin light and heavy chain subunits [63, 64]. Ferritin is involved in iron homeostasis, and iron accumulation is considered a core mediator of ferroptosis. FTH1 and FTL maintain proper cellular iron levels and protect cells from iron overload-related damage [65, 66]. Ketamine may alleviate ferroptosis-induced damage to ovaries and oocytes by regulating the expression of GPX4 and iron-storage proteins FTH1 and FTL, limiting iron accumulation and reducing lipid peroxidation.

This study was conducted using a mouse model, which may not fully recapitulate the complexity of clinical depression in humans. Ketamine possesses well-recognized addictive potential and raises reproductive safety concerns, requiring strict dose control under clinical supervision and safety evaluation. More biological experiments are needed to further verify the causal relationship between ferroptosis and reproductive dysfunction. In addition, our study focused primarily on reproductive function and did not explore the corresponding neurobiological changes, representing another important limitation.

In conclusion, ketamine significantly ameliorated ovarian dysfunction and oocyte quality impairment and alleviated depressive-like behaviors in CORT-induced depressive mice. These protective effects are mediated via modulation of the ferroptosis pathway, as evidenced by the restoration of Fe^2+^, ROS, GSH, and lipid peroxidation homeostasis in oocytes, the attenuation of mitochondrial abnormalities, and the regulation of the ferroptosis-related proteins GPX4, FTH1, and FTL in ovarian tissues. Collectively, these findings highlight a promising therapeutic target for depression-associated reproductive disorders, offering new perspectives for the treatment of infertility accompanied by depressive symptoms.

## Materials and methods

### Animals

Female Institute of Cancer Research (ICR) mice (6–8 weeks of age) were maintained under specific pathogen-free conditions at a controlled temperature of 24 ± 2 °C and relative humidity of 50–60%, with a 12 h light–dark cycle (lights on from 9:00 a.m. to 9:00 p.m.). Mice had ad libitum access to food and drinking water. All animal experiments were approved by the Institutional Animal Care and Use Committee at Xiamen University.

### Experimental design

After a 7-day acclimatization period, mice were randomly divided into four groups [67, 68]. Mice in the control group received saline (vehicle) injections daily for 21 days. Mice in the CORT group received CORT (20 mg/kg) subcutaneous injections daily for 21 days. Mice in the CORT + K10 and CORT + K30 groups received CORT (20 mg/kg) subcutaneous injections daily for 21 days, supplemented with intraperitoneal injections of ketamine (10 mg/kg or 30 mg/kg) once every 7 days. The dosing regimen is shown in Fig. S1A.

### Behavioral tests

Four behavioral tests were conducted as previously described [69]. Briefly, in the OFT, the duration spent in the central area was recorded as an indicator of exploratory behavior. In the EPM, the time spent in the open arms was measured to assess anxiety-like behavior. Behavioral despair was evaluated using the TST and the FST, in which immobility time was quantified. In the TST, immobility was defined as the absence of limb and trunk movement. In the FST, immobility corresponded to the absence of active movements except those necessary to maintain the head above water. Data were recorded using TopScanLite software.

### Serum hormone measurement

Blood samples were collected via retro-orbital plexus puncture, centrifuged to isolate serum, and sent to the Beijing North Institute of Biological Technology (Beijing, China) for hormone measurement. Radioimmunoassay was used to quantify serum concentrations of E2, T, and P. Enzyme-linked immunosorbent assay was used to quantify serum corticosterone, FSH, and LH.

### Histologic analysis of the ovary

The ovarian tissues were fixed, dehydrated, and embedded in paraffin. Serial sections (5 μm thick) were prepared, and five consecutive tissue sections were mounted on a slide. After hematoxylin-eosin staining, the number of follicles was assessed under a light microscope.

### Monitoring of the estrus cycle

The vagina was gently rinsed with 0.9% normal saline, and the rinsing solution was used for preparing the smear. It was fixed with 95% ethanol for 5 min, and then stained with alkaline methylene blue to determine the stage of the estrus cycle. The observation was conducted using an optical microscope (Leica DM6B, Germany). The estrus cycle of mice was divided into four stages: proestrus, estrus, metaestrus, and diestrus [70].

### TEM

The ovarian tissues were fixed in 2.5% glutaraldehyde, followed by three rinses in PB buffer. Samples were subsequently post-fixed in dimethylarsenate buffer (containing 1% osmium tetroxide) and embedded in Araldite resin. Ultrathin sections were double-stained with 3% uranyl acetate and lead citrate, and ultrastructural features were examined using a transmission electron microscope.

### Oocyte and embryo collection

For superovulation, mice received 10 IU pregnant mare serum gonadotropin (PMSG; Ningbo Sansheng Pharmaceutical). GV-stage oocytes were collected 48 h after PMSG administration by manual dissection of the ovaries. For in vivo MII-stage oocytes, an additional 10 IU human chorionic gonadotropin (hCG; Ningbo Sansheng Pharmaceutical) was administered 48 h after PMSG, and oocytes were retrieved from the ampullary region 14 h later. Oocytes were cultured in M2 medium under mineral oil at 37 °C in 5% CO_2_. For embryo collection, female mice were injected with 10 IU PMSG followed by 10 IU hCG after 48 h and paired with males at a 1:1 ratio. The presence of a vaginal plug the following morning was designated as 0.5 dpc. Females were sacrificed by cervical dislocation at 3.5 dpc, and blastocysts were recovered by flushing the uterus with KSOM medium.

### Immunofluorescence

For sample preparation, oocytes and embryos were fixed in 4% paraformaldehyde for 30 min, permeabilized with 0.5% Triton X-100 in PBST for 20 min, and blocked with 1% bovine serum albumin for 1 h. Samples were incubated with primary antibodies overnight at 4 °C, followed by secondary antibody. For live-cell staining, oocytes were incubated with fluorescent probes for 30 min at 37 °C in 5% CO₂, washed three times in M2 medium, and transferred to confocal dishes. Imaging was performed using a Zeiss LSM 880 confocal microscope. The antibodies and probes are described in Tables S1 and S2.

### Western blotting

Ovaries were processed with RIPA lysis buffer (Meilunbio®, Dalian, China) containing protease inhibitors to obtain total protein. The proteins were separated by SDS-PAGE (CINOTOHI®, Changsha, China) and transferred onto a PVDF membrane (Millipore, Bedford, Massachusetts, USA). The membrane was blocked with 5% skimmed milk at room temperature for 1 h, then incubated overnight at 4 °C with antibodies. After washing away excess substances, a secondary antibody was added and incubated. After another wash, the immune-reactive protein bands were detected using the Azure 300 system and the ECL chemiluminescence kit. The antibodies are listed in Table S1, Original WB images in the supplementary file.

### Single-cell transcriptome sequencing analysis

MII oocytes were collected from the Control, CORT, and CORT + K10 groups (15 oocytes per sample, four biological replicates per group), lysed, and stored at −80 °C before sequencing at APTBIO (Shanghai, China). RNA samples were detected based on the A260/A280 absorbance ratio with a Nanodrop ND-2000 system (Thermo Scientific, USA), and the RIN of RNA was determined by an Agilent Bioanalyzer 4150 system (Agilent Technologies, CA, USA). Only qualified samples were used for library construction. Libraries were constructed using the ABclonal mRNA-seq Library Preparation Kit and sequenced on an Illumina NovaSeq 6000 platform. Raw Fastq reads were processed to remove adapters and filter low-quality reads (≥60% bases with quality ≤25 or >5% undetermined bases) to obtain clean reads, and aligned to the reference genome using HISAT2. DEGs were identified using DESeq2 with the criteria of |log_2_ (Fold Change)| ≥ 1 and adjusted *P* < 0.05. GO/KEGG enrichment was performed using the clusterProfiler R package.

### Non-targeted metabolomics analysis

Ovarian samples from the Control, CORT, and CORT + K10 groups (two ovaries per sample, six biological replicates per group) were stored at −80 °C and analyzed by GENWIZ (Shanghai, China) using non-targeted metabolomics. Approximately 100 mg (±2%) of tissue per sample was extracted, processed, and filtered for LC–MS analysis, including quality control procedures. Chromatographic separation was performed using an ACQUITY UPLC® HSS T3 column. Pathway enrichment analysis was conducted using MetaboAnalyst (v5.0), and metabolites with *P* < 0.05, |log₂ (Fold Change)| ≥ 0 were considered significant.

### Statistical analysis

Immunofluorescence intensity was quantified using ZEN 2.0 software (Carl Zeiss Microscopy). Western blot band densities were analyzed using ImageJ (National Institutes of Health) to quantify gray levels. Statistical analyses were performed using ANOVA followed by post hoc LSD test in GraphPad Prism 10 (GraphPad, Inc., USA), assuming normal distribution and similar variance. The sample size for each group is indicated as (*n*) in the corresponding legends. Sample sizes were chosen based on prior experience and conventions in the field. No statistical method was used to predetermine sample size. No samples were excluded from the analysis (unless there was a failure in the sample, such as protein degradation). Data were expressed as mean ± standard deviation (SD). *P* < 0.05 was considered statistically significant. The investigators were not blinded to allocation during outcome assessments. All experiments were performed at least in biological triplicate.

## Supplementary information

Supplemental Material WB

Spplementary File (Fig. S1, Table S1 and S2)

## Data Availability

The data that support the findings of this study are available from the corresponding author upon reasonable request. The raw sequence data reported in this paper have been deposited in the China National Center for Bioinformation/Genome Sequence Archive (subCRA071796).
