# Supplementary material for: Ketamine improves ovarian function and oocyte quality in CORT-induced depression mouse model by ferroptosis pathway
Source: Cell Death Discov. 2026 Aug 8;12:439. doi: 10.1038/s41420-026-03291-9 (PMC13737059; doi:10.1038/s41420-026-03291-9)
Supplement: Supplementary file 1 [file 41420_2026_3291_MOESM2_ESM.docx]

**Supplementary Information**

**Fig. S1**

**(A)** Schematic of the dosing regimen. **(B)** Body weight gain over 21 days (n = 11). (**C)** Statistical analysis of the ovarian coefficient (n = 6). **(D)** Statistical analysis of the number of days in each estrus cycle stage (n = 5). The analysis was conducted over two consecutive estrous cycles. **(E)** Representing images showing the estrous cycle. proestrus (P), estrus (E), metaestrus (M), diestrus (D). ns: not significant.

**Supplementary Table S1. Antibody information**

| **Antibody name** | **Company**  **(Item No.)** | **Dilution**  **ratio** |
| --- | --- | --- |
| Rabbit anti-Histone H3 (di methyl K9) | Abcam (ab1220) | IF; 1:200 |
| OCT4 recombinant rabbit monoclonal antibody | Huabio (SD0750) | IF; 1:200 |
| Rabbit anti-γ-H2A.X (phospho S139) | Abcam (ab2893) | IF; 1:200 |
| anti-α-Tubulin-FITC monoclonal antibody | Sigma (F2168) | IF; 1:250 |
| GPX4 Antibody | Abmart (T56959) | WB; 1:2000 |
| Ferritin Light Chain Rabbit mAb | ABclonal (A11241) | WB; 1:2000 |
| Rabbit Ferritin Heavy Chain Rabbit pAb | ABclonal (A1144) | WB; 1:2000 |
| Alexa Fluor ^TM^ 594 secondary antibody | Invitrogen (A21207) | IF; 1:100 |
| Alexa Fluor® 555 | Huabio (HA1135) | IF; 1:100 |

**Supplementary Table S2. Probes information**

| **Probes name** | **Company (Item No.)** |
| --- | --- |
| One Step TUNEL Apoptosis Assay Kit | Meilunbio (MA0223) |
| Reactive Oxygen Species Assay Kit | Beyotime Biotechnology (S0033) |
| Mitochondrial Membrane Potential Assay Kit with JC-1 | Solarbio (M8650) |
| Annexin V-FITC Apoptosis Detection Kit | Beyotime Biotechnology (C1062) |
| FerroOrange | Dojindo (F374) |
| BODIPY™ 581/591 C11 (Lipid Peroxidation Sensor) | Invitrogen (D3891) |
| Monochlorobimane (Chlorobimane，mBBr) | MedChemExpress (HY-101899) |
